# Supplementary material for: Causal Relationship between Aging and Anorexia Nervosa: A White-Matter-Microstructure-Mediated Mendelian Randomization Analysis
Source: Biomedicines. 2024 Aug 16;12(8):1874. doi: 10.3390/biomedicines12081874 (PMC11351342; doi:10.3390/biomedicines12081874)
Supplement: Supplementary file 1 [file biomedicines-12-01874-s001.zip › Supplementary Figure/Figure S4/Singe SNP plot of MR analysis for TL to ubm-b-1952.pdf]

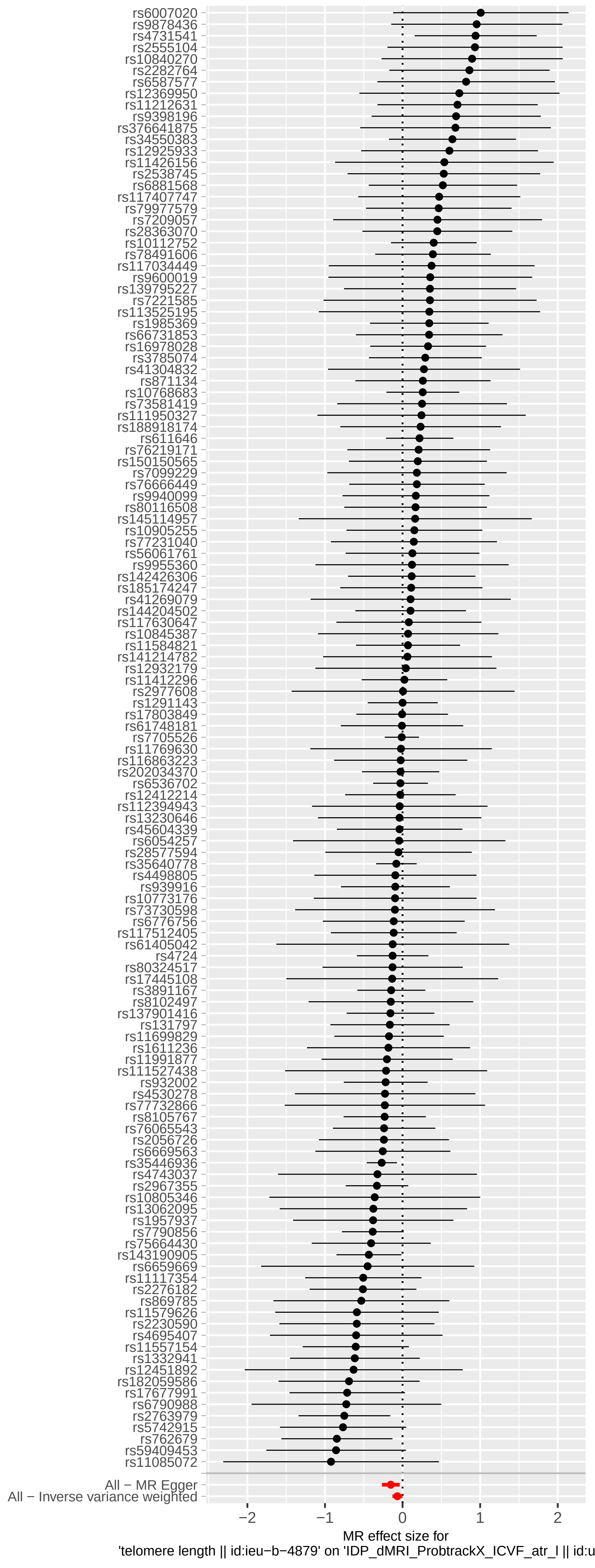
rs6007020
rs9878436
rs4731541
rs2555104
rs10840270
rs2282764
rs6587577
rs12369950
rs11212631
rs9398196
rs376641875
rs34550383
rs12925933
rs11426156
rs2538745
rs6881568
rs117407747
rs79977579
rs7209057
rs28363070
rs10112752
rs78491606
rs117034449
rs9600019
rs139795227
rs7221585
rs113525195
rs1985369
rs66731853
rs16978028
rs3785074
rs41304832
rs871134
rs10768683
rs73581419
rs111950327
rs188918174
rs611646
rs76219171
rs150150565
rs7099229
rs76666449
rs9940099
rs80116508
rs145114957
rs10905255
rs77231040
rs56061761
rs9955360
rs142426306
rs185174247
rs41269079
rs144204502
rs117630647
rs10845387
rs11584821
rs141214782
rs12932179
rs11412296
rs2977608
rs1291143
rs17803849
rs61748181
rs7705526
rs11769630
rs116863223
rs202034370
rs6536702
rs12412214
rs112394943
rs13230646
rs45604339
rs6054257
rs28577594
rs35640778
rs4498805
rs939916
rs10773176
rs73730598
rs6776756
rs117512405
rs61405042
rs4724
rs80324517
rs17445108
rs3891167
rs8102497
rs137901416
rs131797
rs11699829
rs1611236
rs11991877
rs111527438
rs932002
rs4530278
rs77732866
rs8105767
rs76065543
rs2056726
rs6669563
rs35446936
rs4743037
rs2967355
rs10805346
rs13062095
rs1957937
rs7790856
rs75664430
rs143190905
rs6659669
rs11117354
rs2276182
rs869785
rs11579626
rs2230590
rs4695407
rs11557154
rs1332941
rs12451892
rs182059586
rs17677991
rs6790988
rs2763979
rs5742915
rs762679
rs59409453
rs11085072
All − MR Egger
All − Inverse variance weighted
−2
−1
0
1
2
MR effect size for
'telomere length || id:ieu−b−4879' on 'IDP_dMRI_ProbtrackX_ICVF_atr_l || id:u
